# Everyday episodic memory in amnestic mild cognitive impairment: a preliminary investigation

**DOI:** 10.1186/1471-2202-12-80

**Published:** 2011-08-04

**Authors:** Muireann Irish, Brian A Lawlor, Robert F Coen, Shane M O'Mara

**Affiliations:** 1 Mercer's Institute for Research on Aging, St. James's Hospital, Dublin, Ireland; 2 Trinity College Institute of Neuroscience, Trinity College, Dublin, Ireland; 3 Neuroscience Research Australia, Randwick, Sydney, Australia; 4 School of Medical Sciences, the University of New South Wales, Sydney, Australia

## Abstract

**Background:**

Decline in episodic memory is one of the hallmark features of Alzheimer's disease (AD) and is also a defining feature of amnestic Mild Cognitive Impairment (MCI), which is posited as a potential prodrome of AD. While deficits in episodic memory are well documented in MCI, the nature of this impairment remains relatively under-researched, particularly for those domains with direct relevance and meaning for the patient's daily life. In order to fully explore the impact of disruption to the episodic memory system on everyday memory in MCI, we examined participants' episodic memory capacity using a battery of experimental tasks with real-world relevance. We investigated episodic acquisition and delayed recall (story-memory), associative memory (face-name pairings), spatial memory (route learning and recall), and memory for everyday mundane events in 16 amnestic MCI and 18 control participants. Furthermore, we followed MCI participants longitudinally to gain preliminary evidence regarding the possible predictive efficacy of these real-world episodic memory tasks for subsequent conversion to AD.

**Results:**

The most discriminating tests at baseline were measures of acquisition, delayed recall, and associative memory, followed by everyday memory, and spatial memory tasks, with MCI patients scoring significantly lower than controls. At follow-up (mean time elapsed: 22.4 months), 6 MCI cases had progressed to clinically probable AD. Exploratory logistic regression analyses revealed that delayed associative memory performance at baseline was a potential predictor of subsequent conversion to AD.

**Conclusions:**

As a preliminary study, our findings suggest that simple associative memory paradigms with real-world relevance represent an important line of enquiry in future longitudinal studies charting MCI progression over time.

## Background

Mild Cognitive Impairment (MCI) represents a potential transitional stage between non-pathological aging and Alzheimer's disease (AD), referring to individuals who display objectively measured cognitive deterioration in the context of preserved functional capacity and the absence of dementia [1]. Whilst MCI does not necessarily represent a prodrome of AD, mounting evidence suggests that within the subsample of amnestic multi-domain MCI, an elevated risk for progression to dementia exists [2,3]. Although MCI comprises a behaviourally heterogeneous cohort [4], delayed memory testing and recall-based assessments have been identified as the most discriminating factors in flagging those individuals at risk of progression to AD [5,6]. To date, the locus of research has focused on episodic memory processes and their vulnerability in MCI and AD; however, the nature of this episodic deficit in MCI is relatively under-researched [7]. Furthermore, the tests used to assess memory deficits in MCI are often administered as part of large neuropsychological batteries with little relevance for participants' everyday functioning.

One branch of episodic memory that has emerged as particularly promising from a potential diagnostic perspective is that of associative memory [2,8]. Associative memory refers to the linking of component parts, such as words or objects, to create an integrated composite, either directly or via spatial, temporal or other kinds of relationships [9] and represents a fundamental feature of episodic memory capacity. Anecdotal evidence from MCI individuals and their family members suggests that associative memory failures are common, such as recognizing someone but failing to recollect the person's name or where they know the person from [10]. Face-Name associative tasks represent an interesting analogue of the associative encoding individuals are faced with in daily life, and the formation of cross-modal associations between inherently unrelated items of information is likely to be hippocampal-dependent [11]. Furthermore this branch of associative memory appears to be compromised in the earliest stages of AD whereas mixed results have been obtained for MCI individuals [11]. Neuroimaging studies have shown marked reduction in hippocampal and entorhinal cortex volumes in MCI [12], with such pathology at a transitional level to normal aging and AD [13]. Importantly, these medial temporal lobe regions are typically involved in episodic memory [11] with a central role ascribed to the hippocampus for relational or associative memory [14,15]. Associative paradigms, however, are under-recognised in this field, despite previous demonstrations of their ability to separate deteriorating from stable MCI at an early stage [8]. From a clinical standpoint, therefore, the encoding and retrieval of face-name pairs represents an attractive method of investigating potential deficits in associative memory in MCI.

A second branch of episodic memory with direct relevance for everyday functioning that is commonly affected in ageing relates to spatial navigation and way-finding [16]. Spatial navigation refers to the process of determining and maintaining a course or trajectory from one place to another [17] and can be conceptualised as allocentric (i.e., environment based [18]), or egocentric (i.e., self based, [19]). The regions affected earliest in the AD pathological disease process are thought to play critical roles in human navigation [20]. AD patients frequently present with difficulties in spatial orientation in everyday activities [21], often failing to find their way in unfamiliar environments and new spatial settings during travelling and shopping. Despite the importance of spatial memory in everyday functioning, this area is less frequently investigated particularly with respect to MCI and dementia [22]. A number of recent studies have demonstrated impairments in spatial memory in MCI using virtual reality paradigms [23], analogues of the Morris Water Maze task [24], and route-learning tasks [25]. A selective impairment of spatial navigation has been reported in multi-domain amnestic MCI [24], and the authors argue that the disorientation commonly observed in MCI is attributable to impaired spatial memory. They further suggest that if spatial memory begins to decline early in the disease process, presymptomatic measures of spatial navigation may be useful tools to detect individuals at risk of developing dementia prior to the onset of clinical symptoms. Despite mounting evidence for compromised spatial memory in MCI, few neuropsychological studies have examined the extent to which this branch of episodic memory is impaired in this clinical group.

The current picture suggests that episodic memory deficits are strong predictors of future progression to AD [2]. Given that an amnesic syndrome is typically the earliest symptom of AD [11], it is critical to understand the nature of these deficits in MCI. The contribution of the frontal lobes to episodic memory is well established [26], and therefore it is also important to ascertain the degree to which executive functions are compromised in amnestic MCI, as recent studies have argued for the predictive efficacy of such tests for the progression of MCI to AD [4,27]. Exploring the spectrum of episodic memory impairments in MCI affords us the opportunity to investigate the possible clinical utility of episodic memory tasks, particularly those with direct relevance for everyday functioning of participants. A strong rationale exists for using experimental tasks with real-world meaning given that functional decline is the key criterion for diagnosing AD. Accordingly, traditional single-memory tests may not validly contribute to the differential diagnosis of MCI and AD [22]. The aim of this study was to characterise the nature of the memory impairment in MCI using experimental tasks probing multiple domains of episodic memory function. These include associative, spatial and everyday memory tasks that are analogues of real-world scenarios and are commonly encountered by individuals in their daily lives. In addition, we aimed to obtain preliminary data regarding which of these tasks, if any, could potentially serve as an aid to identifying those individuals in the prodrome of AD.

## Results

### Demographics

Controls ranged in age from 69-86 years (age: mean ± SD = 76.0 ± 4.3) and had an average of 14.0 years (± 3.1) in formal education (range: 11-21 years). MCI participants ranged in age from 62-88 years old (age: 71.8 ± 6.8) and spent an average of 13.8 years (± 4.7) in formal education (range: 7.5-25 years). The two groups did not differ for years in education, *F*(1,32) = 0.41, *MSE *= 0.64, *p *= .841, however the MCI group was an average of 4.2 years younger than the control group, *F*(1, 32) = 4.658, *MSE *= 148.53, *p *= .039. Sex was not evenly distributed across the groups, χ^2^(33) = 5.673, *p *= .017, with a female: male ratio of 14:4 in the control group compared with 6:10 in the MCI group. These participant groups have been described in a previous study [28].

### Progression from MCI to AD

At follow-up, 6 of the 16 MCI participants (37.5%) had converted to probable AD (MCIc), and 1 case was diagnosed with behavioural-variant frontotemporal dementia (bvFTD), with the remaining 9 participants representing stable MCI (MCIs). The bvFTD patient was not included in any MCI subgroup analyses. Two participants showed an increase in MMSE scores at follow-up; however, this was not sufficient to denote a return to healthy cognitive functioning, as evidenced by persistent cognitive deficits, with neuropsychological test scores falling below 1.5 standard deviations of age-adjusted norms. Stable MCI cases scored on average 25.9 (± 0.8) on the MMSE in comparison with the converted MCI cases who scored 23.5 (± 2.6) at follow-up. None of the demographic variables were significant predictors of progression to AD (Age: *p *= .367; Education: *p *= .770; Sex: *p *= .886; NART IQ: *p *= .970).

### Acquisition and delayed recall on RBANS

At baseline, MCI participants showed significant deficits in acquisition of new material *F*(1, 32) = 29.509, *MSE *= 392.32, *p *< .0001, and delayed recall, *F*(1,32) = 44.474, *MSE *= 307.06, *p *< .0001), on the RBANS story task compared with controls (Table 1). MCI participants scored on average 6.8 points lower than controls for immediate recall [95% C.I. = 4.25, 9.36] and 6.7 points lower for delayed recall [95% C.I. = 4.18, 7.86].

**Table 1:** Baseline performance across episodic memory subdomains (mean ± SD) for control and Mild Cognitive Impairment participants.

	Assessment	Controls (n = 18)	MCI (n = 16)	Group Effect
Acquisition and	RBANS immediate recall	20.6 (2.7)	13.7 (4.5)	***
Delayed Recall	RBANS delayed recall	9.8 (2.0)	3.8 (3.2)	***
Associative Memory	Face Name Trials 1-4	4.2 (1.1)	1.8 (0.5)	***
	Face Name delayed	4.3 (1.7)	1.7 (1.9)	***
	Face Name free recall	5.6 (0.7)	2.8 (1.9)	***
Spatial Memory	Landmark Recognition	4.8 (0.9)	3.7 (2.1)	n/s
	Landmark Location	5.0 (1.5)	4.4 (1.5)	n/s
	Pointing task errors°	114.6 (62.5)	150.2 (53.1)	n/s
	Route Description	13.3 (2.1)	10.5 (3.5)	**
	HR Recall	8.4 (3.2)	5.5 (3.0)	*
	HR Recognition	7.7 (1.3)	6.9 (1.5)	n/s
	HR Temporal Order	16.4 (9.4)	25.0 (6.5)	**
Everyday Memory	MMQ "Yesterday"	23.8 (0.4)	21.0 (2.9)	***
	MMQ "1 week ago"	19.4 (5.7)	12.1 (6.7)	**
	EMQ total score	23.6 (14.0)	62.7 (32.2)	***

* *p < .05*; ** *p < .01*; *** *p < .001; *n/s, non-significant, MCI = Mild Cognitive Impairment, HR = Hospital Route task, MMQ = Mundane Memory Questionnaire, EMQ = Everyday Memory Questionnaire

**Figure 1:**
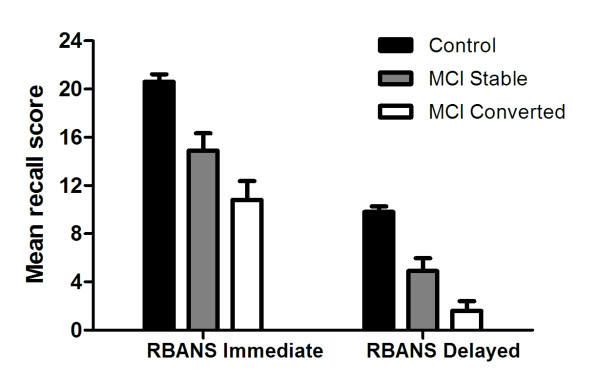
Baseline RBANS story performance for controls, stable MCI and converted MCI subgroups (Bars represent 95% C.I.)

A Mann-Whitney *U *test revealed a significant difference between the MCI subgroups for delayed recall on the RBANS story task, *U *= 10.5, *p *= .036. MCIc participants recalled on average 3.5 details less on their baseline RBANS assessment in comparison to MCIs participants [95% C.I. = .4, 6.2] (see Figure 1).

### Acquisition of face-name pairs

Mann-Whitney *U *tests revealed significant deficits in the MCI group across all face-name learning trials (*p *< .0001). Wilcoxon Signed Ranks tests revealed a significant difference between Trials 1 and 3 (*p *= .006) in the MCI group, with recall of face-name pairs peaking in Trial 3. In the control group, significant differences following Bonferroni corrections (corrected alpha = .0125) were found between Trials 1 and 3 (*p *= .001), Trials 1 and 4 (*p *= .004), Trials 2 and 3 (*p *= .004) and Trials 2 and 4 (*p *= .002). This reflected the increase in learning across trials. Not surprisingly, controls scored almost at ceiling on the fourth learning trial of the Face-Name pairs task with 50% of the sample scoring the maximum 6 points (Mean score: 5.1, SD = 1.2). Of interest here, however, was the performance of controls versus patients on the delayed recall subscale of this task (see below).

A significant difference between the MCI subgroups was found on Trial 2 of the Face-Name pairs task, *U *= 7.0, *p *= .015, with MCIs participants recalling on average 2.2 face-name pairs in comparison with 0.8 pairs recalled by the MCIc group (Mean Difference = 1.4; 95% C.I. = .4, 2.8).

### Delayed recall of associative items versus associative pairings

**Figure 2:**
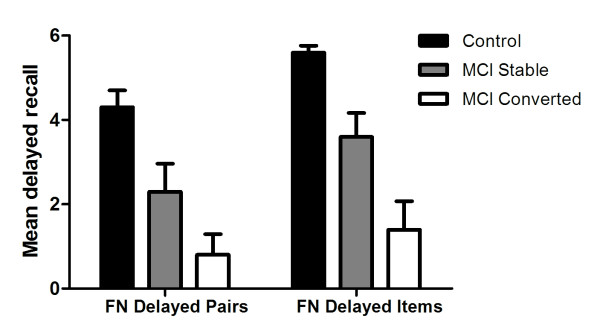
Delayed associative pairings and item recall on the Face Name task at baseline for controls, stable MCI and converted MCI subgroups (FN = Face Name; Bars represent 95% C.I.)

Mann-Whitney *U *tests showed that controls scored significantly higher than the MCI group for delayed pairings (*U *= 46.5, *p *< .001; Mean Difference = 2.5; 95% C.I. = 1.2, 3.8) and delayed recall of test names (*U *= 31.0, *p *< .0001; Mean Difference = 2.7; 95% C.I. = 1.6, 3.8, Figure 2). Importantly, controls did not show evidence of a ceiling effect on the delayed recall subscale with only 6 out of 18 controls (33.3%) scoring the maximum 6 points. No significant difference between the MCIs and MCIc subgroups was present for delayed recall of face-name pairs (*p *= .089), however the MCIc subgroup performed significantly worse than the MCIs subgroup on delayed associative item memory (*U *= 8.0, *p *= .017; Mean Difference = 1.5; 95% C.I. = -.3, 3.3).

Wilcoxon Signed-Rank tests revealed a significant difference between Face-Name pairs test subscales for controls with higher delayed recall of names (Mean score: 5.6) in comparison with face-name pairings (Mean difference = 1.3; *Z *= -2.725, *p *= .003; 95% C.I. for difference = .5, 2.0). This effect was also evident in the MCIs group, (Names: 3.9, Face-Name Pairs: 2.4; *Z *= -2.401, *p *= .008; 95% C.I. for mean difference = .4, 2.2). However, for MCIc participants, this effect was attenuated, *Z *= -1.732, *p *= .042, with less pronounced differences between the average number of names recalled (1.3) versus the average number of correctly identified face-name pairs (0.8) [95% C.I. for difference = -.1, 1.3].

### Spatial memory performance

A MANOVA revealed a marginally significant difference between MCI and control participants for Landmark Recognition, *F*(1, 32) = 4.102, *MSE *= 10.07, *p *= .051. No significant group differences were evident on the Landmark Location task, *F*(1,32) = 1.436, *MSE *= 3.31, *p *= .240. No significant group effects were observed on the Pointing Task *F*(1,32) = 3.778, *MSE *= 24.87, *p *= .061. In contrast, the Route Description task dissociated between participant groups *F*(1, 32) = 8.434, *MSE *= 68.00, *p *= .007, with controls scoring on average 2.8 points higher than MCI participants [95% C.I. = .85, 4.82]. Controls scored significantly higher than MCI participants for immediate recall on the Hospital Route task, *F*(1, 32) = 7.152, *MSE *= 70.69, *p *= .012 (Mean Difference = 2.9; 95% C.I. = .69, 5.09). No significant differences between the participant groups were present for Hospital Route Recognition *F*(1, 32) = 3.071, *MSE *= 6.08, *p *= .089. The MCI group exhibited significantly poorer accuracy in reconstructing the correct temporal order of photographs from the Hospital Route *F*(1, 32) = 9.365, *MSE *= 628.10, *p *= .004, (Mean Difference = 8.6; 95% C.I. = 2.88, 14.34). Mann-Whitney *U *tests did not reveal any significant differences between the MCIs and MCIc subgroups for any of the spatial tasks (all *p *> .3).

### Everyday memory recall

Controls scored significantly higher than the MCI group for the recall of events that occurred "Yesterday", *U *= 55.5, *p *< .001 (Mean Difference = 3.0; 95% C.I. = 1.6, 4.5), and "Yesterday 1 week ago", *U *= 46.5, *p *< .001 (Mean Difference = 7.8; 95% C.I. = 3.4, 12.3) on the MMQ. No significant differences were found between MCIs and MCIc subgroups for MMQ recall for "Yesterday" (*U *= 22.0, *p *= .350) and "Yesterday 1 week ago" (*U *= 25.0, p = .500). Wilcoxon Signed-Rank tests revealed that controls recalled significantly more detail for "Yesterday" compared with "Yesterday 1 week ago", (Z = -3.189, *p *= .001; Mean Difference: 4.4; 95% C.I. = 1.6, 7.2). Similarly, both MCI subgroups recalled more details for events in closer temporal proximity to the present day (MCIs: *Z *= -2.431, *p *= .015, Mean difference = 9.5; 95% C.I. = 4.2, 14.8; MCIc: *Z *= -2.214, *p *= .027, Mean difference = 8.6; 95% C.I. = 5.7, 11.6). On the Everyday Memory Questionnaire (EMQ), MCI participants rated their lapses in memory as significantly higher than control participants, *F*(1, 32) = 17.95, *p *< .0001, (Mean difference = 3.0; 95% C.I. = 1.6, 4.4), however, Mann-Whitney *U *tests failed to reveal any differences in EMQ ratings between stable and converted MCI subgroups (*U *= 22.0, *p *= .356).

### Executive Function

At baseline, a MANOVA revealed that MCI participants were impaired on the Digit span backwards task (*p *= .044, Mean difference = 1.5; 95% C.I. = .1, 3.0), Trails B-A (*p *= .005, Mean difference = 45.7; 95% C.I. = 19.9, 71.6), Category fluency (*p *< .0001, Mean difference = 12.3; 95% C.I. = 6.4, 18.2) and the Stroop task (*p *= .012, Mean difference = 20.7; 95% C.I. = 5.2, 36.1) in comparison with controls [28]. Mann-Whitney *U *tests failed to reveal any significant differences between MCIs and MCIc participants on any of the tests of executive function at baseline (all *p *> .3).

### Exploratory prediction of conversion to AD

**Figure 3:**
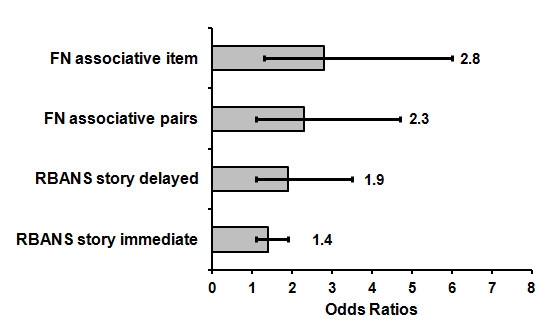
Odds ratios from exploratory individual regression analyses using Face Name and RBANS story experimental tasks as predictors for conversion from MCI to AD (FN = Face Name, Bars represent 95% C.I.)

The tasks which differentiated between controls and MCI participants at baseline, and between MCIs and MCIc subgroups at follow-up, were the Face-Name Pairs task and the RBANS story recall task. These tasks were included as predictor variables in a series of exploratory independent regression analyses. Figure 3 shows the individual predictive power of the four test variables of interest (Face-Name delayed name recall and delayed face-name pairs recall, and RBANS story immediate recall and delayed recall), ranked in ascending order according to the magnitude of their odds ratios. Delayed recall of names on the Face-Name pairs task emerged as the best predictor of conversion to AD in this sample (Associative item memory; *p *= .008; Odds ratio = 2.8, 95% C.I. = 1.3, 6.0; Nagelkerke R square = .564), with the regression model correctly classifying 88% of participants. This was followed by delayed face-name pairings (Associative pairs memory; *p *= .030; Odds Ratio = 2.3, 95% C.I. = 1.1, 4.7; Nagelkerke R square = .378), the RBANS story delayed recall (*p *= .032; Odds Ratio = 1.9, 95% C.I. = 1.1, 3.5; Nagelkerke R square = .563), and the RBANS story immediate recall (*p *= .011; Odds Ratio = 1.4, 95% C.I. = 1.1, 1.9; Nagelkerke R square = .466).

**Figure 4:**
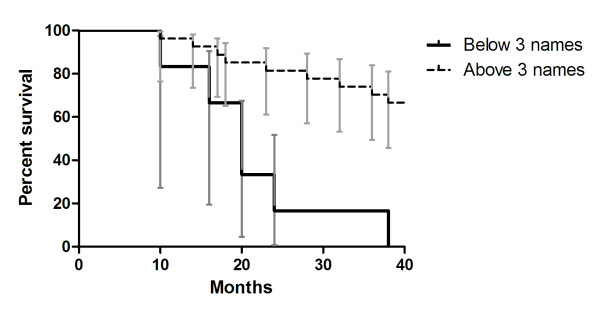
Exploratory cumulative survival function using Face-Name task delayed associative item recall to predict conversion from MCI to AD (Bars represent 95% C.I.)

Figure 4 shows the cumulative survival function across time for MCI and control participants (bvFTD patient excluded, n = 33) scoring above and below the Face-Name associative item memory threshold score of 3. Visual examination of score distributions on scatterplots was used to select the threshold score of 3 for the Face-Name associative item memory subscale. The resulting regression model revealed that the Face-Name associative item memory threshold variable was a significant predictor of conversion to AD in the regression equation (*β *= -1.092, *p *= .012) with an odds ratio of 3.0 [95% C.I. for odds ratio = 1.3, 7.0]. Using this cut-off score of below 3 on the Face-Name associative item memory subscale achieved a sensitivity of 1.0 and a specificity of .86.

## Discussion

The objective of this study was to investigate the nature of the episodic memory impairment in MCI using experimental tasks with direct impact on everyday functioning and relevance for patients in their everyday lives (e.g., story-telling, remembering faces and names, spatial navigation, and everyday memory). MCI participants were significantly impaired across all domains of episodic memory assessed, with the greatest baseline deficits evident on episodic memory tests of acquisition, delayed recall and associative memory. Longitudinal follow-up data suggested that delayed associative memory performance at baseline may have some predictive utility for subsequent conversion to probable AD. This intimates that a simple Face-Name pairs test may have the potential to be a useful neuropsychological task for identifying individuals in the prodromal stage of AD.

### Exploratory findings: Potential predictive power of Face-Name pairs task

The most significant finding to emerge from this study concerns the associative memory deficits evident in the MCI group, which were detectable at baseline using a simple Face-Name pairs task. Of note for future studies are our preliminary findings using a cut-off score of 3 names on the delayed associative item subscale of the Face-Names pairs task at baseline for the prediction of conversion from MCI to probable AD within two years. Critically, the percentage of converted MCI participants in our sample mirrors the conversion rates previously reported [29] allowing us some confidence in our results. The predictive efficacy of the Face-Name pairs task (88%) is encouraging given that multivariate combinations of episodic memory tasks with executive function, recognition memory, visuospatial memory processing speed and visuospatial episodic memory tests have demonstrated lower classification accuracy than our current findings (84% [4]). Our data build on the current view that associative episodic memory tests represent a critical line of enquiry in MCI [2,30]. We suggest here that there is considerable scope to incorporate episodic memory tasks with real-life relevance for participants into future larger-scale longitudinal studies, with the goal of arriving at an optimal diagnostic battery that can successfully identify those MCI individuals in the prodrome of clinically probable AD.

While delayed associative item performance was notably impaired in MCI, significant episodic memory impairments were also detectable for the acquisition of face-name pairs across trials and the subsequent retention of these real-world pairings. This finding is in contrast with recognition and forced-choice face-name associative paradigms which have not revealed differences between MCI and control participants [11]. The existence of encoding and retention difficulties in MCI points towards potential neurodegenerative changes in the medial temporal lobe regions subserving memory formation [31], as well as posterior cingulate regions [32], and possibly more widespread gray matter loss [33], with medial temporal lobe regions no longer able to activate during attempted learning [11]. The observation of higher scores for delayed recall of names (associative item) compared with delayed recall of face-name pairs (associative pairs) pointed towards a differential profile of associative deficits in controls and MCI participants. Controls' performance supported the Associative Deficit Hypothesis [34], which specifies that older adults exhibit a unique deficit for associations over and above their deficits, if any, in item memory. Importantly, on the delayed associative subscale, only 6 out of 18 controls (33.3%) correctly remembered all 6 Face-Name pairs, ruling out the possibility of ceiling effects in this sample. Interestingly, both MCI subgroups showed a similar pattern to controls albeit at a lower level, however the deficit for associations was attenuated in the converted MCI subgroup and may reflect a floor effect. We suggest the converted MCI subgroup's impairments are an artefact of associative encoding difficulties, precluding the formation of an association between face and name, and retention deficits which were evident at delayed recall. This dovetails nicely with reports that MCI individuals with learning and retention deficits at baseline show a significantly higher likelihood of developing AD over two years [33].

### Acquisition of material in MCI

Further deficits in the encoding and retention of new material were evident on the RBANS story task, with MCI participants displaying significant impairments in immediate and delayed story recall. This mirrors the common complaint of MCI individuals not remembering stories that are recently relayed and suggests impaired executive functioning [35]. Indeed, performance on the Trail Making Test was inversely correlated with RBANS delayed recall (*p *< .0001). Using percentage retention scores [36], the aggregated MCI sample at baseline obtained a retention percentage of 55% in comparison with 96% for controls. Interestingly, the stable MCI subgroup retained 72% of the story in comparison with 28% for the converted MCI subgroup, corroborating optimal diagnostic accuracy indices of scores < 50% retention to distinguish AD from MCI [36].

### Impaired spatial memory in MCI

This study builds on previous findings of disrupted spatial memory in MCI during route encoding [25], on an analogue of the Water maze task [24], and a comparison of real-world versus virtual-reality spatial navigation [23]. Consistent with previous studies using similar methods [25], we have confirmed that spatial memory, across a number of domains, is disrupted in MCI. Critically using a novel combination of real-world measures and pencil and paper tasks, we found a dissociation between those tasks requiring actual navigation, either mental (Route Description) or physical (Hospital Route tasks), and those which relied predominantly on recognition processes (Landmark Recognition and Location) or mental rotation (Landmark Pointing). Importantly, by including a longitudinal component, we could investigate the possible predictive efficacy of spatial memory tasks for conversion to AD. The only tasks that dissociated between MCI and control participants at baseline were those which required engaging in navigation. This finding is consistent with previous reports which suggest that actual navigation recruits different neural regions than table-top arrays or pencil and paper tasks [37]. The geographical disorientation found on the Route Description task in the MCI group occurred despite the provision of cardinal directions and a clear map of the imaginary city. This finding is important, as similar decrements in route description have been observed in the mild stages of AD and these deficits are attributed to allocentric disorientation [38].

For real-world navigation on the Hospital Route task, MCI individuals showed significant impairments for immediate recall and temporal ordering, the latter of which likely relies on the integrity of the frontal lobes [39] and may reflect frontal lobe dysfunction in MCI of a greater magnitude than in non-pathological ageing. Although recognition of stimuli encountered on the Hospital Route task was intact in MCI, it has been noted [25], that landmark recognition likely engages neural regions that are lost in more advanced stages of AD such as more posterior medial temporo-occipital regions [40]. The finding of impaired spatial navigation and spatial memory in MCI is worthy of further investigation using a larger, sex-balanced sample, as it is possible that the poor predictive power of spatial tasks is due to the relatively small numbers in the present study. Importantly, whilst sex was not evenly distributed between the participant groups, the over-representation of males in the MCI group did not mask the significant spatial memory deficits found in this cohort compared with controls. The relative sparing of spatial recognition memory in MCI warrants further attention, particularly where the functional capacity of the individual is concerned, given that topographical disorientation is often one of the initial manifestations of AD [38,41] and likely contributes to the compromised capacity to carry out such activities of daily living as shopping and travelling unaccompanied.

### Everyday mundane memory and insight in MCI

In keeping with studies documenting compromised retrieval of past events in MCI [13,28], everyday memory for the previous day and week was impaired in our MCI cohort. At baseline, MCI participants exhibited significant deficits in retrieving contextual details for the "mundane" events of the previous day and one week prior, such as failing to remember the clothes they wore, where they had been, and who they had met. In a previous study [28], recall of contextual details from autobiographical memories (ABMs) across the lifespan, including an experimentally-verified event that occurred one week previously, was found to be compromised in this MCI cohort. Successful recall of the minutiae of the previous day and week may rely more on overgeneral or semanticised schemas that are relatively preserved in contrast with contextually rich episodic ABMs mediated by medial temporal lobe structures known to be affected in MCI [13]. Of interest is the finding of significantly higher subjective ratings of everyday memory lapses in the MCI group compared with controls, indicating a degree of insight into their deficits. The level of awareness for memory functioning has been shown to vary in MCI [42], whereas lack of insight into deficits is consistent with the typical presentation of patients with AD [43]. Further investigation of these differences in self-awareness is therefore warranted, as this may represent an important distinction between MCI and AD patient groups, as impaired awareness of one's deficits makes an important contribution to functional decline [44,45]. Additionally, how the everyday episodic memory deficits demonstrated here impact on the daily activities of the MCI patient is an interesting, but unresolved, question worthy of further investigation. It is likely that the impairments across the domains of encoding, associative, spatial and mundane memory impact on the everyday activities of the MCI individual. This represents an important area for future research, particularly in terms of elucidating how such episodic memory deficits relate to behavioural changes reported by the patient and/or their informant, or whether these changes relate to carer stress. Whether such deficits are amenable to cognitive retraining is a further issue of critical importance.

### Executive function in MCI

Despite the apparent preservation of insight into their deficits, the MCI group showed significant impairments on tests of executive function at baseline (Category Fluency, Trails B-A, and Stroop, see [28], suggesting difficulties with set-switching and response inhibition. Given the importance of the frontal lobes in spatial and non-spatial episodic memory [46,47] it is not surprising that the MCI group exhibited significant decrements on many of the memory tasks used in this study. However, no significant differences were present between the MCIs and MCIc subgroups for baseline performance on the executive tasks. In addition, these tasks did not predict conversion to AD. This finding may reflect the purposive selection of multi-domain MCI participants, which may truncate the MCI sample by excluding individuals with mild executive deficits, such that any potential relationships between the test variables are obscured [48]. It would therefore be worthwhile to investigate the combined predictive power of associative episodic memory tasks and measures of executive function in this respect.

A number of limitations should be considered in light of the current findings, primarily the small sample from which we base our conclusions. While the emergence of significant findings in a sample this small provides encouraging preliminary findings, it is possible that our results are specific to the present sample and may not generalise to all cases with MCI. Future studies are clearly warranted to investigate these effects in a larger sample of both single-domain and multi-domain amnestic MCI cases, allowing us more confidence in the generalisablity of the current findings. The interval between baseline and follow-up varied across participants, although a regression analysis showed that time elapsed between baseline and follow-up testing did not predict conversion to AD. Control participants were not followed up longitudinally as part of this study as the emphasis was on identifying important prodromal markers of AD in MCI. It will be important for future studies to follow controls longitudinally to garner further data regarding age-related changes in healthy individuals. While it is unlikely that any of the controls in the present sample were in the early stages of a dementing process, given that changes in cognition are typically detectable from 5 to 6 years prior to diagnosis of AD [49], longitudinal follow-up data for controls would allow us greater confidence in the regression predictions, particularly in the cases of highly educated and high-functioning individuals. Finally, structural neuroimaging data should be incorporated into future longitudinal studies of everyday episodic memory to investigate the degree of hippocampal atrophy in the MCI participants as an index of disease trajectory, which is in itself predictive of rapid conversion to AD [50].

## Conclusions

In summary, this study has explored the decline of episodic memory in amnestic MCI across the real-world domains of story acquisition and recall, face-name pairs association, spatial memory and navigation, and mundane everyday memory. The MCI patients who subsequently converted to AD exhibited a specific profile of episodic memory deficits that was detectable at the baseline visit, notably on tests of delayed associative recall. Using longitudinal data, we found that the delayed associative item subscale of the Face-Name pairs task may hold some predictive utility as a diagnostic aid within the clinical setting. While these results need to be replicated in longitudinal studies with larger samples and follow-up data for controls, our current results support the view that episodic memory tasks with real-world relevance are important to assist in the identification of MCI individuals who may convert to AD within a relatively short period of time. It is crucial to identify MCI individuals who are likely to convert to AD in the very early stages, facilitating swift intervention with anticholinergic drugs, for which the earlier the treatment the better the long-term outlook [51]. The creation of a neuropsychological test that can reliably predict those MCI cases that will progress to AD remains the "holy grail of neuropsychology" [52]. Furthermore, the implementation of neuropsychological assessments that can be understood by the patient as having relevance to their everyday life is imperative. Based on this preliminary study, a simple associative memory task such as the encoding and retention of face-name pairs represents a promising line of enquiry for future investigations.

## Methods

### Participants

#### MCI Recruitment

A sample of 16 unmedicated individuals with a diagnosis of amnestic MCI were recruited through the Mercer's Institute for Research on Aging (MIRA) memory clinic and diagnosed by a consultant-led multi-disciplinary team using the consensus criteria from the International Working Group on Mild Cognitive Impairment [1] and the MCI Working Group of the European Consortium on Alzheimer's disease criteria [53]. The diagnostic work-up included an interview with the individual and a reliable informant covering current and past personal and medical history, current health and mental status, physical and neurological exam, routine laboratory screen, ratings of functional status and behaviour, and neuropsychological testing. MRI or CT brain scan was done as a routine part of the work-up. The consensus-based multi-disciplinary team diagnosis of amnestic MCI was conferred if the individual's memory deficits fell at least 1.5 standard deviations below age-adjusted normative scores on at least one standardised test of memory including the Cambridge Cognitive Examination CAMCOG [54] and the Delayed Word Recall (DWR) test [55,56] in the context of preserved functional capacity. In the case of MCI individuals with high pre-morbid ability who did not score at least 1.5 S.D. below age-adjusted norms on these tests, the Wechsler Memory Scale Immediate and Delayed Logical Memory sub-tests (WMS-III; [57]) and the Rey Osterrieth Complex Figure test (Copy and recall; [58]) were also included to ensure that memory deficits were present. Further neuropsychological tests for all MCI patients included verbal fluency (FAS test) and semantic category fluency [59], and the Boston Naming Test - 30 item [60].

The memory deficits exhibited by the MCI participants were subjectively reported by the MCI individual and corroborated by an informant. Emphasis was placed on the preserved functional capacity of the individual, which was determined based on informant report using the Instrumental Activities of Daily Living scale (IADL) and the Personal Self Maintenance Scale (PSMS) [61]. Where basic activities of daily living were preserved and complex instrumental functions were either intact or minimally impaired, the patient was deemed to be functionally capable. Specifically, they did not fulfil criteria for dementia. The majority of MCI participants in this sample were diagnosed with multi-domain MCI (n = 13), exhibiting deficits in memory and executive functioning, whilst the remaining 3 participants received a diagnosis of single-domain MCI, with deficits residing exclusively within the domain of memory [62]. MCI cases were recruited for this study within 6 months of their diagnosis to ensure that the data collected were representative of patients' current disease state.

#### Control Recruitment

Eighteen controls were sourced from a local Active Retirement Association, and were screened prior to taking part to exclude for any neurological complaints, a prior serious head injury, history of psychiatric illness or alcohol abuse.

#### General Cognitive Screening

Prior to administration of the experimental tasks, general cognitive screening for all participants (MCI and controls) included the Mini-Mental State Examination (MMSE, [63]), the Clock Drawing Test (10 point scoring system, [64]), the National Adult Reading Test (NART, [65]), the Geriatric Depression Scale - 15 item version (GDS-15, [66]) and the Instrumental Activities of Daily Living scale (IADL, [61]). Controls scoring < 27 on the MMSE or < 8 on the Clock Drawing Test were excluded, as were any participants scoring > 7 on the GDS-15, which was taken as indicative of pervasive depression (see [67]). Furthermore, all controls scored within normal limits on the Word List Memory subtest from the Consortium to Establish a Registry for Alzheimer's disease (CERAD - Word List; [68]). Ethical approval was obtained from the St. James's Hospital and Adelaide and Meath Research Ethics Committee and all participants gave informed consent prior to testing in accordance with the Declaration of Helsinki (1991).

### Assessment of episodic memory

#### Episodic acquisition

The "Story memory" component from the immediate memory index of the Repeatable Battery for the Assessment of Neuropsychological Status (RBANS) [69] was used to assess acquisition and learning. A short story describing a house fire, containing 12 key details, was read aloud to participants over two trials, and participants were asked to recall the story in as much detail as possible immediately following each encoding trial. This led to a total maximum score of 24 for Immediate Recall (2 × 12). Delayed recall of the story was assessed 15 minutes following encoding (maximum score of 12). Scoring was based upon verbatim recall with one point awarded for each detail correctly retrieved.

#### Associative memory

A modified Face-Name pairs task [70] was used comprising 6 black and white face stimuli (hair included) and 6 accompanying first names presented across four encoding blocks. Participants were shown each face stimulus on an A4 page with its corresponding first name adjacent to the picture, one at a time, at a rate of one every 2 seconds and were asked to commit the names to memory under intentional learning conditions [34]. At recall, participants were shown the face stimuli in a new randomised order and asked if they could remember the corresponding name for each face. This encoding and immediate recall procedure continued across 4 trials. One point was awarded for each correctly named face, with a maximum score of 6 points per trial. A constructional praxis distraction task was completed to prevent rote rehearsal between encoding and recall blocks. All stimuli were randomised to prevent order effects and to ensure the association between the face and name was being recalled. Delayed recall was assessed 30 minutes following encoding using the face stimuli as cues in a newly randomised order (Maximum Score = 6). Participants were told they would see the facial stimuli again and were asked to remember the correct name for each face. Free recall was assessed at the end of the Face-Name test session by asking participants to recall as many names as they could without being able to see the facial stimuli. This provided information regarding the associative encoding of the name stimuli versus the associative binding of faces-name pairs.

#### Landmark recognition and location

To gauge participants' familiarity with Dublin city, prior to the administration of the Landmark Pointing test (see below), participants were required to identify six well-known landmarks from Dublin city, from colour photographs of the canonical view of each landmark [71]. Participants were shown each photograph one at a time and asked which well-known Dublin landmark was depicted. One point was awarded for each correct answer (Recognition Max score = 6). An additional point was awarded if participants could accurately locate each landmark on a blank map of Dublin City Centre with all spatial information omitted. Participants were shown the 6 colour photographs one at a time and asked to point to the corresponding letter on the map denoting the correct location for the landmark. The only defining features on the map included the River Liffey dividing the city into North and South and the letters A-F, which represented the location of the six different landmarks (Location Max score = 6).

#### Landmark pointing task

Participants stood on a large laminated compass affixed to the floor, facing 0 and pointed to the six landmarks from the Landmark Recognition task. They were instructed to imagine themselves standing in front of one of the six landmarks and to point in the direction of each of the remaining five landmarks in turn, as though giving directions to somebody unfamiliar with the city [72]. This was repeated across three trials originating from three different starting locations in the city centre. The accuracy of pointing was established by dropping a plumbline from the participant's finger to the compass on the floor and the experimenter read off the corresponding angle from the compass. The degree of error was calculated by subtracting participants' measurements from the correct set of angles taken by the experimenter using Google Earth, with an average error score calculated across the three starting points for each participant.

#### Route description task

A black and white map of a virtual city was presented, and participants were instructed to give directions to someone going from point × to point Y describing the best possible route [71]. The map contained a series of streets and buildings with no defining features. Four letters were used to signify the location of four landmarks (A, B, C, D), and the compass points of North, South, East, West were included as a reference. Participants were asked to narrate their chosen route aloud and could trace the route with a pencil or finger to show the experimenter their trajectory. The experimenter documented each response verbatim. Each route was broken down into a number of requisite steps, generating maximum points of 9, 5, and 6 points for routes A to B, B to C, and C to D, respectively (Maximum score = 20). Where a participant deviated from the correct route, or provided ambiguous directions, no points were awarded for those directions. One mark was awarded for each correct step, irrespective of whether an incorrect step occurred at any point in the sequence.

#### Hospital route navigation task

A real-life analogue of topographical memory was designed to test participants' free recall of a route they physically traversed within the hospital grounds, with one change of floor and one change of building. The experimenter accompanied participants on the route and narrated the course of the journey, pointing towards specific proximal landmarks, and highlighting changes of direction, turns, and floor changes. Immediate free recall of the route was tested without any prompts (Route Acquisition) and scored according to 15 steps including direction, building and floor changes, and salient landmarks (Max score = 15). For Route Recognition, participants were shown 10 colour photographs comprising 5 "correct" and 5 "lure" stimuli and were awarded 1 point for each correct answer, i.e. correctly identifying a target stimulus and correctly rejecting a lure stimulus (Max score = 10). Route Temporal Order was assessed by asking participants to organise 10 photographs from the traversed route, according to the order in which they were encountered. The absolute difference between the participants' order and the correct temporal order was calculated, with higher scores reflecting poorer performance on the task.

#### Everyday memory

The Mundane Memory Questionnaire (MMQ) [71] is a twelve-item questionnaire which probes the recall of a "typical" day, covering two time periods, "Yesterday" and "Yesterday One Week ago". Participants are asked a series of questions in a structured format, which follows the temporal order of a "typical" daily routine from morning to evening (e.g. getting dressed, travelling to work, the weather that day, receiving letters, meeting friends, eating lunch, shopping, reading, work around the house, watching television, making phone calls that evening). In this study, the MMQ was modified by changing work-related questions to reflect everyday items such as leaving one's house and means of transport that day. The questions proceeded as follows, "Do you recall getting dressed on this day? If so, what did you wear?" One point was awarded for a "Yes" response for each question with an additional point awarded for provision of details specific to that question (Max score per time period = 12 × 2 = 24). No points were awarded for a "No response" indicating failure to recall, or the failure to provide specific details. The MMQ was originally validated in a sample of 422 healthy adult volunteers and has been shown to demonstrate good overall reliability (Cronbach's alpha = .85) [71].

The Everyday Memory Questionnaire [73] was also administered to participants as an index of subjective awareness of memory lapses that have occurred over the preceding 3 months. Participants were asked to read 28 statements pertaining to various memory problems, and indicate the frequency with which they had noticed that particular memory lapse, ranging from "Not at all in the last 3 months" (0 points) to "More than once a day" (9 points). Higher scores were indicative of greater memory problems with possible scores ranging from 0 to a maximum score of 252 (= 28 × 9).

#### Executive Function

To fully understand the extent of executive dysfunction in the MCI group, all participants completed the following tests; (1) Digit Span and Spatial Span subtests of the Wechsler Memory Scale III [57]; (2) Controlled Oral Word Association Test (COWAT; CFL [74]); (3) Category Fluency (fruit, vegetables, animals); (4) Trail Making Test Parts A and B [75]; (6) Stroop Test (pencil and paper version) [76]. These data have been reported previously [28].

#### Follow-up of MCI participants

As part of their ongoing clinical care, all MCI participants re-attended the MIRA memory clinic, approximately within two years of the baseline visit (Mean time elapsed 22.4 months ± 9.5). This resulted in an attrition rate of 0 for follow-up data. In keeping with the diagnostic procedure described above, all MCI participants were re-tested using the same neuropsychological test battery and the same diagnostic work-up was undertaken, but MRI/CT were not routinely repeated. The experimental everyday memory tasks were not re-administered at follow-up. Informants were interviewed to obtain a history of decline and loss of function (if any) for the patient over the intervening time period. The multi-disciplinary team reviewed each case and decided whether the patients represented (a) progression to dementia (APA 1994), which depended particularly on whether or not there was evidence of functional decline on standardised ratings of functional status; (b) if progressed to dementia, was it clinically probable AD based on NINCDS-ADRDA criteria (i.e. scores on neuropsychological tests falling below 2 SD compared to age-adjusted norms) [77]; (c) stable MCI based on the Winblad et al. [1] and Portet et al. [53] criteria for MCI; or (d) an improvement from baseline.

### Statistical Analyses

Multivariate analyses of variance (MANOVA) were used to investigate group differences at baseline (Control, MCI) across demographic and screening variables, with nonparametric tests used for non-normally distributed variables. Ninety-five % confidence intervals (CI) were calculated and Bonferroni corrections were used for multiple comparisons. Backwards multiple regression models were run to investigate the predictive effect of demographic and screening variables for conversion to AD. Converted MCI (MCIc) were compared to stable MCI (MCIs) cases using nonparametric Mann-Whitney *U *tests. Sex was included as a covariate in all between-group analyses. A series of binary logistic regression analyses for all participants combined were run for each variable of interest to determine odds ratios and 95% C.I.s for potential predictors of conversion to AD. Nagelkerke R-square values were calculated for each of the regression analyses, as an additional confirmatory estimate of the relative importance of the memory measures as potential predictors of conversion to AD. A Cox-Exploratory regression survival analysis was also run for all MCI and control participants combined (bvFTD patient excluded, n = 33) as an exploratory step to investigate potential predictors of conversion to AD at follow-up.

## Competing interests

The authors declare that they have no competing interests.

## Authors' contributions

MI designed the study, performed all experimental testing and data collection, conducted all data analyses and interpretation, and wrote the manuscript. BA oversaw the execution of the study and helped to revise the manuscript. RC helped to design the study, assisted with participant recruitment, contributed to data analysis and interpretation, and helped to draft and revise the manuscript. SOM helped to design the study, facilitated access to specific test batteries, and assisted with interpretation of results and revising the manuscript. All authors have read and improved the final manuscript.
